# Pretilachlor Releasable Polyurea Microcapsules Suspension Optimization and Its Paddy Field Weeding Investigation

**DOI:** 10.3389/fchem.2020.00826

**Published:** 2020-10-22

**Authors:** Hongjun Chen, Xiu Liu, Shuqi Deng, Hongkun Wang, Xiaoming Ou, Linya Huang, Jingbo Li, Chenzhong Jin

**Affiliations:** ^1^Hunan Provincial Key Laboratory of Fine Ceramics and Powder Materials, School of Materials and Environmental Engineering, Hunan University of Humanities, Science and Technology, Loudi, China; ^2^Key Laboratory of Pesticide Harmless Application in Hunan Higher Education, Hunan Provincial Collaborative Innovation Center for Field Weeds Control, Hunan University of Humanities, Science and Technology, Loudi, China; ^3^Forestry Bureau of Lanshan County, Lanshan, China; ^4^National Engineering Research Center for Agrochemicals, Hunan Research Institute of Chemical Industry, Changsha, China

**Keywords:** pretilachlor, weeding, herbicidal activity, polyurea microcapsules suspension, response surface methodology

## Abstract

In this study, pretilachlor was encapsulated into polyurea microcapsules prepared by water-initiated polymerization of polyaryl polymethylene isocyanate and eventually made into pretilachlor microcapsules suspension (PMS). We used response surface methodology (RSM) combined with the Box–Behnken design (BBD) model to optimize the formulation of PMS. The encapsulation efficiency (EE) of PMS was investigated with respect to three independent variables including wall material dosage (X_1_), emulsifier dosage (X_2_), and polymerization stirring speed (X_3_). The results showed that the regression equation model had a satisfactory accuracy in predicting the EE of PMS. To achieve an optimal condition for PMS preparation, the dose of wall material was set to 5%, the dose of emulsifier was set to 3.5% and the polymerization stirring speed was set to 200 rpm. The EE of PMS was up to 95.68% under the optimized condition, and the spherical shape with smooth surface morphology was observed. PMS was also proven to have delayed release capability and *in vivo* herbicidal activity against barnyard grass [*Echinochloa crusgalli* (L.) Beauv.] with an LC_50_ value of 274 mg/L. Furthermore, PMS had efficient weed management compared to commercially available 30% pretilachlor emulsifier (PE), showing a promising potential application for weeding paddy fields.

## Introduction

Weeds are recognized as major biological constraints that hinder the attainment of optimal rice productivity (Smith, 1968). Currently, chemical herbicides have been used extensively throughout the world to control weeds, prevent crop decline, and enhance productivity (Heap, 2014). As a pre-emergent herbicide [2-chloro-2,6-diethyl-N-(2-propoxyethyl) acetanilide], pretilachlor has effective herbicidal activities against weeds in rice fields by preventing plant cells and algae from generating long-chain fatty acids (Diyanat et al., 2019). However, pretilachlor is usually toxic to mammals and fish and even has high phytotoxicity in crops such as rice (Kaushik et al., 2006; Takahashi et al., 2007; Diyanat et al., 2019). The overuse of pretilachlor may cause harm to the environment by damaging non-target plants, water, sediment, and food (Ismail and Handah, 1999; Vencill et al., 2012; Palma et al., 2014; Papadakis et al., 2015). For these reasons, numerous efforts have been devoted to minimizing the adverse effects and environmental toxicity of herbicides, such as using them along with safeners, packing them with carrier materials, or loading them into microcapsules (Hedaoo et al., 2013; de Oliveira et al., 2015; Cao et al., 2016; Liu et al., 2016; Wu et al., 2020). Microcapsules with controlled release abilities can prevent herbicides from leaking via evaporation or degradation during application so as to ultimately improve the herbicidal efficiency and minimize non-target toxicity (Hedaoo et al., 2013, 2014; Zhang et al., 2016, 2018), which has become a popular and feasible trend in recent years. In light of this, the design and synthesis of microcapsule systems with high herbicide retention rates and appropriate sustained-release ability are of practical value for agricultural herbicide formulations.

Nowadays, several methods such as phase separation, *in situ* polymerization, layer-by-layer polyelectrolyte deposition, and interfacial polymerization, have been proposed to prepare a microencapsulation system with controlled release performance (Scarfato et al., 2007). The microcapsule shell wall can be prepared with suitable prepolymer by a suitable synthetic method, which is crucial to the final drug loading efficiency and the release performance of the microcapsule suspension (Kumar et al., 2016; Yoo et al., 2017). A variety of polymerization agents, such as polyamide, polyethylene, polyurethane, and polyurea, have been used to prepare microcapsules by interfacial polymerization technology (Scarfato et al., 2007; Hedaoo et al., 2014; Jia et al., 2014; Florez-Grau et al., 2016). For instance, pretilachlor loaded polycaprolactone nanocapsules (PCL) were synthesized and demonstrated with significant herbicide activity and lower cytotoxicity, suggesting promising prospective applications in environmentally friendly PCL-herbicide systems construction (Diyanat et al., 2019). Pretilachlor encapsulated polyethylene glycol nanospheres were proved to perform better than that of the commercially available pretilachlor formulation (Kumar et al., 2016). It has been reported that the microcapsules synthesized by the chemical reaction between isocyanate groups and nucleophiles (such as amines, alcohols, and water) are less costly and environmentally friendly (Scarfato et al., 2007; Hedaoo et al., 2013, 2014; Zhang et al., 2016). Currently, isocyanates prepolymers such as isophorone diisocyanate (IPDI), 2,4-toluene diisocyanate (TDI), 4,4′-diphenylmethane diisocyanate (MDI), hexamethylene-1,6-diisocyanate (HMDI), and polyaryl polymethylene isocyanate (PAPI) were jointly used with organic chain extenders [such as urea, 1,4-butanediol, diethylenetriamine and hexamethylene-1,6-diamine (HMDA)] to prepare polyurea microcapsules (Scarfato et al., 2007; Takahashi et al., 2008; Hedaoo et al., 2013, 2014; Jia et al., 2014; Ma et al., 2017; Christian and Wagh, 2018; Li et al., 2019). For example, pretilachlor polyurea microcapsule prepared with HMDI and HMDA in the n-octane solvent was demonstrated to have release abilities (Christian and Wagh, 2018). In addition, metolachlor polyurea microcapsules were first synthesized through TDI and urea in water with excellent encapsulation efficiency (81.45%). It is worth noting that although organic chain extenders urea was used, no organic solvent was applied during the metolachlor polyurea microcapsule suspension synthesis, which might prevent the organic solvents from polluting the ecological environment (Takahashi et al., 2008). To the best of our knowledge, pretilachlor herbicide microcapsules prepared by isocyanate polymerization without the addition of organic chain extenders are insufficient. Consequently, research on the optimization of synthetic parameters that affect the efficiency of herbicide encapsulation is urgently needed.

The interfacial polymerization technique is a suitable method to load herbicide into polyurea microcapsules (Li et al., 2009; Hedaoo et al., 2014). However, several process variables can affect the herbicide encapsulation efficiency of the formed microcapsules. Response surface methodology (RSM) is a set of mathematical and statistical techniques that are commonly used to design experiments, build models, evaluate influencing factors, and find the best conditions for expected response factors (Aslan and Cebeci, 2007; Almeida et al., 2019; Yousefi et al., 2019). Box-Behnken design (BBD) is a type of response surface design that does not include embedded factor or partial factor, which can provide conclusions and detailed information through the interaction of a small number of experiments and operating parameters on all responses (Ferreira et al., 2007; Prakash Maran et al., 2013; Bouriche et al., 2019). BBD as applied in response surface methodology (RSM), constitutes an important design tool for the optimization of different processes. In order to prepare high performance herbicide encapsulated microcapsules, it is of great importance to figure out how the applied variables can influence the encapsulation efficiency.

In this study, water-initiated polymerization of polyaryl polymethylene isocyanate was adopted to prepare pretilachlor polyurea microcapsulate suspension (PMS) with a controlled release function, using a simple interfacial polymerization technique. RSM in conjunction with BBD was applied to establish the functional relationships between three operating variables [wall material dosage (X_1_), emulsifier dosage (X_2_) and stirring speed for polymerization (X_3_)] and the pretilachlor encapsulation efficiency (EE) of PMS, as well as to develop a mathematical model for prediction and determination of optimum conditions for reaching the maximum encapsulation efficiency. The microcapsule particle size, physicochemical stability, surface morphology, and pretilachlor release properties were investigated. Greenhouse experiments were also performed to verify the herbicidal activity of PMS against the barnyard grass [*Echinochloa crusgalli* (L.) Beauv.]. Finally, the as-prepared PMS was used for weeding in paddy fields and we evaluated its efficiency in managing weeds.

## Materials and Methods

### Material and Chemicals

The technical pretilachlor (purity = 95%) was obtained from Shandong Qiaochang Chemical Co., Ltd. (Shandong, China). Commercially available 30% pretilachlor emulsifier containing fenclorim (30% PE) was purchased from Hefei Xingyu Chemical Co., Ltd. (Anhui, China). The wall material polyaryl polymethylene isocyanate (PM 200, industrial grade) was purchased from Wanhua Chemical Group Co., Ltd. (Shandong, China). The sodium salt of alkyl naphthalene sulfonate formaldehyde polymer (MF) was purchased from Molbase Chemical Mall (Shanghai, China). High-boiling-point aromatic solvents (S-200) and Tween-60 (T-60) were purchased from Jiangsu Haian Petroleum Chemical Plant (Jiangsu, China). Fenclorim, xanthan gum, and dimethylbenzene were purchased from Aladdin Biochemical Technology Co., Ltd. (Shanghai, China). All of the methanol (chromatographic grade) was purchased from Tianjin Kemiou Chemical Reagent Co., Ltd. (Tianjin, China). All of the reagents used in this experiment were of analytical reagent grade and were used without further purification. Ultrapure water obtained from an Eped-Plus-E3 system (18.2 MΩ·cm) was used in the study.

### Single-Factor Preparation of Pretilachlor Microcapsules Suspension (PMS)

The PMS (100 g for each batch) was prepared by an interfacial polymerization technique in an oil-in-water emulsion. Through the single-factor test, some experimental parameters affecting the pretilachlor encapsulation efficiency of PMS were studied, such as emulsifier dosage, wall material dosage, and polymerization stirring speed. Generally, 25 g of pretilachlor was dissolved in S-200 (6.0 g) solvent, followed by the addition of a certain amount of wall material PM-200 and emulsifier T-60 to generate a uniform organic phase. Simultaneously, 2.0 g of MF was dissolved in 49 mL of water while stirring to produce a homogeneous aqueous phase. Then, the organic phase was poured into the water phase, and vigorously stirred at 2,500 rpm for 15 min to produce an O/W emulsion. Afterward, the acquired emulsion mixture was stirred at the corresponding speed to trigger polymerization at 35^o^C for 1.5 h, 45^o^C for 1 h, and 55^o^C for 1.5 h. Subsequently, 10 g of 10% xanthan gum aqueous solution was poured into the former solution and stirred for 1 h to obtain the final PMS.

### Estimation of Pretilachlor Encapsulation Efficiency in PMS

Identification of pretilachlor was addressed by comparison of the high-performance liquid chromatography (HPLC) retention times with corresponding standards and co-chromatography with added standards. The HPLC analysis was performed on a Shimadzu chromatographic system (LC-10AVP PLUS), equipped with a UV-Vis detector (SPD-M10Avp). Regarding the HPLC analysis, a solution composed of methanol/water (80:20) was used as the mobile phase, and a reverse-phase Inertsil C-18 column (WondaSil C18-WR, 4.6 × 250 mm, 5 μm, Japan) was applied for separation. Twenty microliter of the sample was injected into the chromatography column, the flow rate of the mobile phase was set to 1 mL/min, and the UV-vis detector was used to collect the 235 nm signal. All experiments were repeated three times. For the pretilachlor content quantification, a calibration curve was obtained by plotting the peak area responses vs. spiked concentrations (0.01, 0.05, 0.1, 0.5, and 1.0 mg/mL). The constructed five-pointed calibration curve (*n* = 3) was linear in the working range: 0.01–1 mg/mL, the function relation was *y* = 2201460*x*+2692 (*R*^2^ = 0.9995), with limits of detection (LOD) 0.0035 mg/mL (Supplementary Figure 1).

The pretilachlor encapsulation efficiency in PMS was evaluated by HPLC analysis. Briefly, 0.5 g of as-synthesized PMS sample was dispersed in 3.0 mL of water and centrifuged for 10 min at a rate of 10,000 r/min to collect precipitate. The centrifugal treatment and precipitation collection were performed twice. The collected precipitate was then dissolved with methanol and sonicated for 15 min to destroy microcapsules, and ultimately diluted with methanol to 25 mL in a volumetric flask. Finally, the pretreated solution was filtered with a 0.45 μm organic membrane for HPLC analysis to obtain the mass of loaded pretilachlor. The mass of pretilachlor was calculated with a standard sample. The encapsulation efficiency (EE) can be calculated according to equation 1:

(1) $$\begin{array}{l} EE\left(\%\right)=\frac{mass\;of\;loaded\;pretilachlor}{mass\;of\;total\;pretilachlor}\times 100 \end{array}$$

### Box-Behnken Experimental Design

Response surface methodology (RSM) was applied to obtain the appropriate preparation parameters of PMS with the highest pretilachlor encapsulation efficiency. A Box-Behnken statistical design (BBD) involving 3 factors, 3 levels, and 17 runs was adopted to optimize research. Table 1 lists all independent variables and dependent variables, and batches were prepared according to the experimental procedures discussed previously. The experimental design consists of a set of points located at the midpoint of each edge and the replicated center point of the multidimensional cube. The investigated independent variables (factors) included wall material dosage (X_1_), emulsifier dosage (X_2_), and polymerization stirring speed (X_3_). The medium levels were wall material dosage (4.0%), emulsifier dosage (3.0%), and polymerization stirring speed (250 r/min), respectively. The pretilachlor encapsulation efficiency of the synthesized PMS (Y) was taken as dependent variables (responses). Table 2 shows the experimental design matrix generated by the software and the corresponding dependent variables. The interaction of independent variables and responses were generated using the following quadratic mathematical model (Equation 2):

(2) $$\begin{array}{l} \text{Y}={\text{b}}_{0}+{\text{b}}_{1}{\text{X}}_{1}+{\text{b}}_{2}{\text{X}}_{2}+{\text{b}}_{3}{\text{X}}_{3}+{\text{b}}_{1,\;2}{\text{X}}_{1}{\text{X}}_{2}+{\text{b}}_{1,\;3}{\text{X}}_{1}{\text{X}}_{3}\\ \;+{\text{b}}_{2,\;3}{\text{X}}_{2}{\text{X}}_{3}+{\text{b}}_{1,\;1}{\text{X}}_{1}^{2}\;+{\text{b}}_{2,\;2}{\text{X}}_{2}^{2}+{\text{b}}_{3,\;3}{\text{X}}_{3}^{2} \end{array}$$

where Y is the dependent variable, b_0_ is the intercept, bi is the estimated regression coefficient for each factor, and X_1_, X_2_, and X_3_ represent the coded independent variables.

**Table 1:** Variables employed in Box–Behnken design.

**Independent variable/factor**	**Levels**		
	**−1**	**0**	**1**
X_1_: wall material dosage (%)	3	4	5
X_2_: emulsifier dosage (%)	2	3	4
X_3_: stirring speed (r/min)	200	250	300
Dependent variable/response	Constraints		
Y: Encapsulation rate (%)	Maximize		

**Table 2:** Box-Behnken design consisting of experiments for the study of three experimental factors in coded and actual levels with experimental results.

**Run no**.	**Coded variables**			**Process variables**			**Actual EE (%)**	**Predicted EE (%)**
	**X_**1**_**	**X_**2**_**	**X_**3**_**	**X_**1**_**	**X_**2**_**	**X_**3**_**		
1	−1	−1	0	3.00	2.00	250	89.89	89.97
2	1	−1	0	5.00	2.00	250	91.72	92.34
3	−1	1	0	3.00	4.00	250	89.62	89.00
4	1	1	0	5.00	4.00	250	95.15	95.07
5	−1	0	−1	3.00	3.00	200	90.48	90.92
6	1	0	−1	5.00	3.00	200	95.54	95.43
7	−1	0	1	3.00	3.00	300	90.19	90.30
8	1	0	1	5.00	3.00	300	94.68	94.24
9	0	−1	−1	4.00	2.00	200	92.85	92.33
10	0	1	−1	4.00	4.00	200	93.26	93.44
11	0	−1	1	4.00	2.00	300	91.84	91.66
12	0	1	1	4.00	4.00	300	91.78	92.30
13	0	0	0	4.00	3.00	250	94.72	94.01
14	0	0	0	4.00	3.00	250	93.75	94.01
15	0	0	0	4.00	3.00	250	93.90	94.01
16	0	0	0	4.00	3.00	250	94.09	94.01
17	0	0	0	4.00	3.00	250	93.59	94.01

### Microcapsules Characterization

The particle size distribution of the PMS was characterized by a laser particle size analyzer (Easysizer 20; Omec Instruments Co., Ltd., Guangdong, China), with D_10_, D_50_, and D_90_ representing the particle sizes at 10, 50, and 90%, respectively. The sample was diluted with water to a suitable concentration and analyzed for 5 parallel measurements to obtain a statistical particle size. PMS was dropped on the slide glass and dried naturally for appearance observation using an optical microscope [BH200, Sunny Optical Technology (Group) Co., Ltd., Zhejiang, China] in 10 × 40 magnification. The morphological appearance of PMS was observed using a scanning electron microscope (FEI Inspect F50, USA). The PMS was diluted with water to a suitable concentration, dropped on the copper mesh, and dried naturally for SEM scanning. After the PMS was diluted with water to a suitable concentration, the Zeta potential was measured by Zetasizer (3000SH, Malvern Instruments Co., Ltd., UK).

### Storage Stability and Release Kinetics

In order to investigate the physicochemical stability, the prepared PMS was packed in glass tubes and stored at 0, 25, and 55^o^C. Then, the pretilachlor content and encapsulation efficiency were evaluated. The amount of pretilachlor released from the PMS was quantified according to a method similar to the quantitative detection procedure for pretilachlor encapsulation efficiency. Briefly, 0.5 g of PMS sample was dispersed in 3 mL of water and then centrifuged for 10 min at a rate of 10,000 r/min to collect precipitate. The total content of pretilachlor remaining in the microcapsules (*M*_∞_) was quantitatively analyzed by HPLC. Then, the precipitate was dispersed in 3 mL of solution (pH 4.0), continuously shaken at 25^o^C at certain intervals, and centrifuged at a rate of 10,000 r/min to collect the precipitate and quantify the release of pretilachlor (*M*_*t*_). The release kinetics of the microcapsules were measured with the Korsmeyer–Peppas model (Ritger and Peppas, 1987a,b; Wu et al., 2020), which is described according to Equation 3:

(3) $$\begin{array}{l} \frac{M_{t}}{M_{\infty }}=Kt^{n} \end{array}$$

Where *M*_*t*_ represents the amount of pretilachlor released in time *t, M*_∞_ represents the amount of pretilachlor released within infinite time, *K* represents the kinetic release constant, and *n* represents the release exponent.

### Herbicide Activity Assays

Barnyard grass was selected as the research object to evaluate the herbicidal activity of PMS. The cultural experiments of potted plants were carried out as follows. Firstly, three quarters of the soil was filled into plastic test pots (9 × 6 × 8 cm), and 15 newly germinated barnyard grass seeds were sowed in each pot. From the next day, 1.5 mL of PMS (150, 300, 600, 1,200, and 2,400 mg/L) with accurate concentrations was sprayed. At the same time, Tween 80 solution (0.1% v/v) was treated as the blank control. After spraying, the barnyard grass seeds were cultivated in an artificial climate box (In the day time: 14 h, 28°C, 80% RH; At night time: 10 h, 25°C, 75% RH). Each treatment was repeated three times for testing. After 14 days of poisoning treatment, the mortality and growth index of barnyard grass were evaluated. The mortality of barnyard grass can be evaluated using Equation 4:

(4) $$\begin{array}{l} Mortality\;\left(\%\right)=\frac{N_{con}-N_{tre}}{N_{con}}\times 100\% \end{array}$$

Where *N*_*tre*_ and *N*_*con*_ denote the number of barnyard grass treated with toxic reagent and blank control, respectively.

### Field Experiment

The field trials were conducted in a test field (11205E, 27406N) in Songshan Village, Shuangfeng County, Hunan Province (Supplementary Figure 2). The experimental site was characterized by red topsoil on flat terrain with a pH of 6.4 and a moderate total organic carbon content. The main weed in the field was barnyard grass. On the 7th day after rice transplantation, a certain ridgeline was constructed to produce experimental plots (2 × 3 m), with a water depth of 3–5 cm in each compartment. The paddy field has been treated with herbicides for nearly a month. After the seventh day, a certain amount of herbicides mixed with sand were sprayed evenly into the test field. It was a cloudy day when the experiment was carried out, with an average temperature of about 35°C. Regarding the dose of herbicide, PMS treatments were assigned to four concentrations of 540, 750, 1,080, and 1,800 g (a.i.)/ha as the test groups, femclorim (the safener agent) shared with PMS [1,800 g (a.i.)/ha], and commercially available PE [540 g (a.i.)/ha] was denoted as the comparison, the blank treatment was sand. After the herbicide treatment, all compartments were treated with conventional management. At regular intervals (7, 14, and 30 days) after application, the amount of barnyard grass in each compartment was collected. By investigating the zigzag five-point rice samples collected in each compartment, the rice growth indexes in each compartment, including plant height and till number, were statistically evaluated. Rice production was also calculated.

### Statistical Analyses

Response surface analysis was performed using Design-Expert software (Trial Version 10.0.4) to ensure the model fitting. Theoretical optimal synthetic parameters were generated from response surface analysis and mathematical models to prepare PMS with the maximum pretilachlor encapsulation efficiency. All data were statistically analyzed using SPSS software (version 22.0) and were expressed in the form of mean ± standard error.

## Results and Discussion

### Screening of Preliminary Experimental Parameters for RSM Modeling

**Scheme 1:**
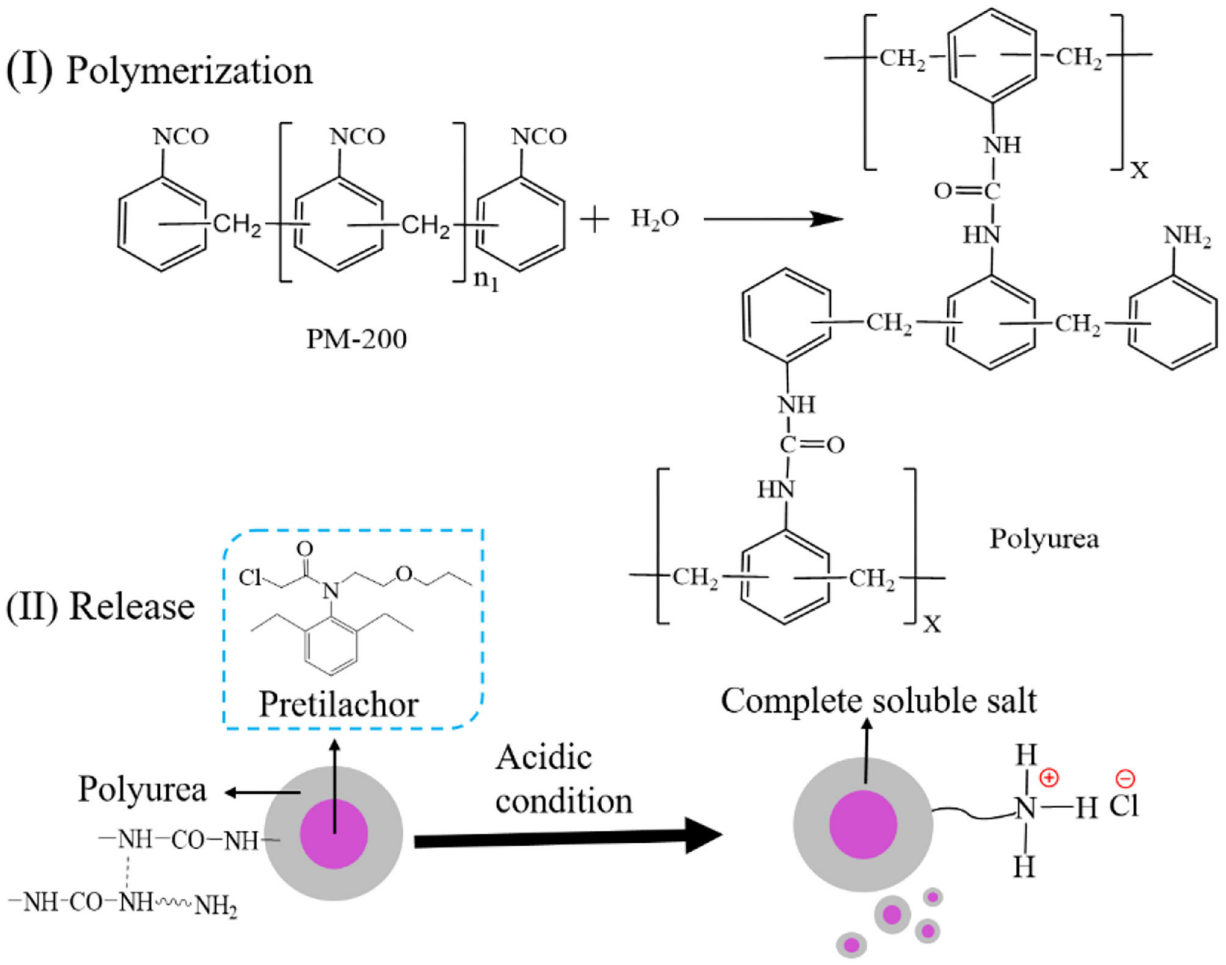
(I) The general polymerization reaction between PM-200 and H_2_O, (II) release mechanism of polyurea PMS microcapsules in acidic condition.

**Figure 1:**
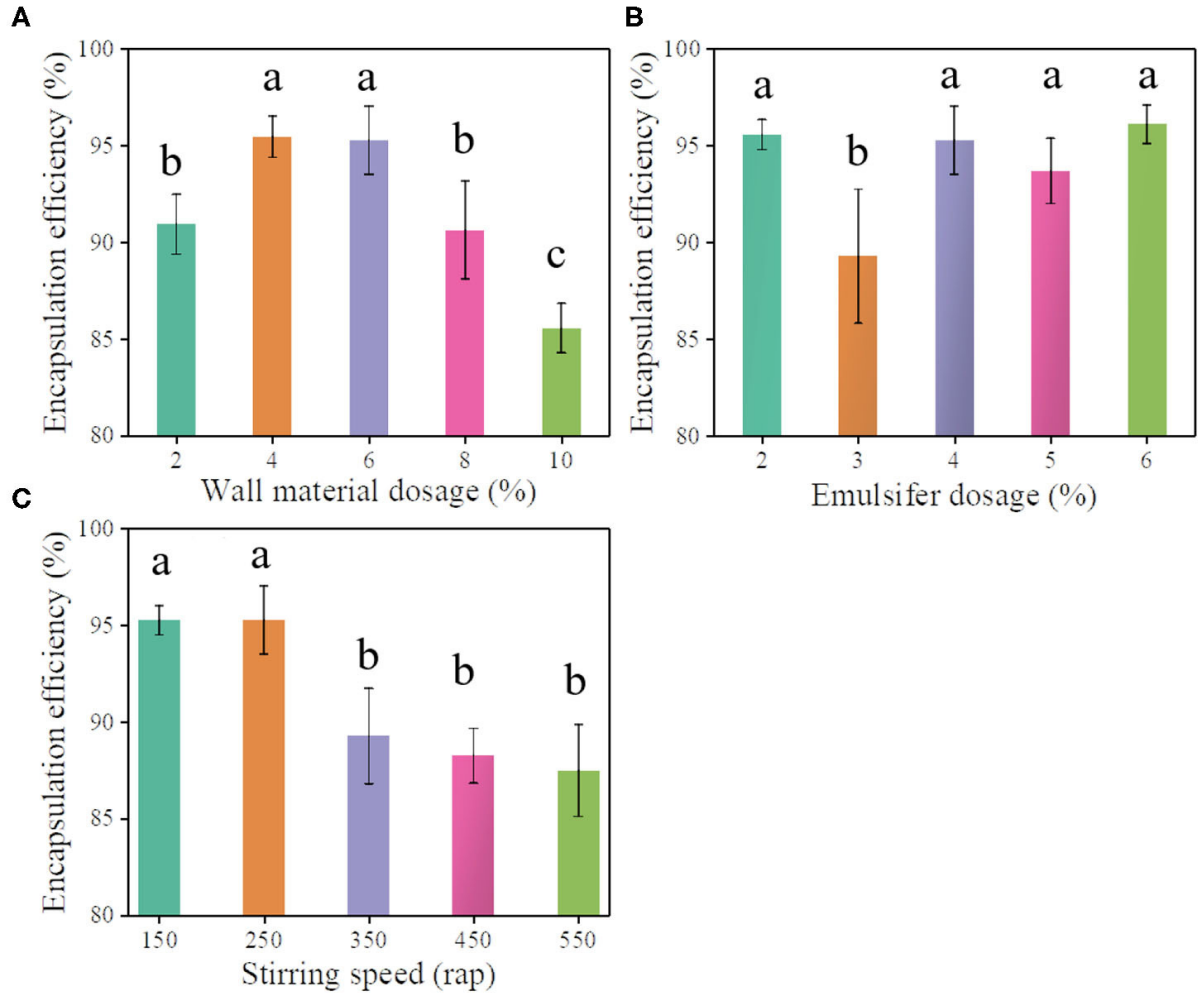
Pretilachlor encapsulation efficiency of PMS prepared under different doses of wall material PM-200 **(A)**, the dose of emulsifier T-60 **(B)**, and stirring speed **(C)**, respectively (Note: Data marked with different lowercase letters are significantly different at the *p* < 0.05 level by Duncan's multiple-range test).

The general interfacial polymerization reaction between PM-200 and H_2_O to generated a polyurea wall of PMS is illustrated in Scheme 1. In brief, interfacial polymerization was initiated by heating the oil-in-water emulsion, where the isocyanate monomers react with H_2_O at the interface (slow step) to form amines, and in turn, react with unhydrolyzed monomers at the interface to form the polyurea microcapsule wall (Takahashi et al., 2008). The choice of the midpoint of independent variables in the Box-Behnken experiment design is crucial to the success of response surface analysis (Ferreira et al., 2007). Before response surface modeling experiments, a series of single-factor experiments were performed to screen out the relative independent variables that affect the pretilachlor encapsulation efficiency of PMS, including wall materials (PM-200) dosage, emulsifier (T-60) dosage, and polymerization stirring speed. As shown in Figure 1A, the obtained encapsulation efficiency gradually increased to the highest level and then turned to decrease at the point when PM-200 usage increased to 4%. As shown in Figure 1B, as the T-60 dose was increased to 3%, the encapsulation efficiency decreased significantly, and then increased to a relatively stable level, which was equal to the level collected at 2%. Considering the encapsulation efficiency changed the most when the dosage was about 3%, to decline the cost of PMS production, therefore the midpoint of T-60 was selected as 3% for RSM investigation. As shown in Figure 1C, the collected encapsulation efficiency increased to the highest level and started to decrease when the polymerization stirring speed increased to 250 rpm. It can be seen that these three parameters had profound relationships with the pretilachlor encapsulation efficiency of PMS. Hence, the midpoint of the dose of PM-200 was set to 4%, that of the dose of T-60 was set to 4% as well, and that of stirring speed was set to 250 rpm for RSM modeling experiment.

### Data Analyses and Validation of the Applied Model on the Encapsulation Efficiency

In this study, Box–Behnken design (BBD) was applied to optimize PMS preparation using an interfacial polymerization technique. Analysis of variance (ANOVA) was used to evaluate the contribution degree of the factors on the pretilachlor encapsulation efficiency (EE%). Values of probability *p* < 0.05 and 0.01 indicate that model terms are significant and highly significant, respectively, and that the *p* > 0.05, which indicates that the model terms are not significant (Almeida et al., 2019). The transformed values of all three experimental factors in coded and actual levels with observed and predicted EE are presented in Table 2. Analysis of variance (ANOVA) for RSM affecting the EE of PMS is shown in Table 3. The EE ranged between 89.62 and 95.54 (Table 2), indicating the EE was affected by the variables. The EE (dependent variable) obtained at various levels of the 3 independent variables (X_1_, X_2_, and X_3_) was subjected to multiple regression to yield a second-order polynomial equation (final equation in terms of coded factors):

(5) $$\begin{array}{l} \text{EE}\left(\text{\%}\right)\;=\;94.01+2.22{\text{X}}_{1}+0.44{\text{X}}_{2}-0.46{\text{X}}_{3}+0.92{\text{X}}_{1}{\text{X}}_{2}\\ \;-0.14{\text{X}}_{1}{\text{X}}_{3}-0.12{\text{X}}_{2}{\text{X}}_{3}-1.06{\text{X}}_{1}^{2}-1.35{\text{X}}_{2}^{2}-0.22{\text{X}}_{3}^{2} \end{array}$$

The quadratic model was the best-fitting model with an insignificant lack of fit (0.1487) and the maximum adjusted *R*^2^ (0.8997), and predicted *R*^2^ (0.4864). The coefficient of determination (*R*^2^) of the model for EE was 0.9561, with an adjusted *R*^2^ of 0.8997, indicating that 95.61% of the model can be predicted. The fitting model showed an *F*-value of 16.95 and a *p*-value of 0.0006 (Table 3), indicating the model was significant. Independent variables X_3_ showed negative coefficients, indicating that high stirring speed is unfavorable to pretilachlor loading. In contrast, X_1_ and X_2_ as well as the interaction between X_1_X_2_ showed positive coefficients, indicating they have a favorable effect on the EE. Among the tested variables in this study, X_1_, X_1_X_2_, ${\text{X}}_{1}^{2}$, and ${\text{X}}_{2}^{2}$ coefficients were significant model terms as the *p* < 0.05.

**Table 3:** Analysis of variance for response quadratic model Encapsulation efficiency (%).

**Source**	**Sum of Squares**	**df^α^**	**Mean square**	***F*-value**	**Prob > F**
Model	56.17	9	6.24	16.95	0.0006^**^
X_1_	35.74	1	35.74	97.07	<0.0001^**^
X_2_	1.54	1	1.54	4.18	0.0801
X_3_	1.66	1	1.66	4.50	0.0716
X_1_ X_2_	3.42	1	3.42	9.29	0.0186^*^
X_1_ X_3_	0.081	1	0.081	0.22	0.6529
X_2_ X_3_	0.055	1	0.055	0.15	0.7101
${\text{X}}_{1}^{2}$	4.75	1	4.75	12.91	0.0088^**^
${\text{X}}_{2}^{2}$	7.70	1	7.70	20.92	0.0026^**^
${\text{X}}_{3}^{2}$	0.21	1	0.21	0.58	0.4716
Residual	2.58	7	0.37		
Lack of fit	1.81	3	0.60	3.15	0.1484
Pure error	0.77	4	0.19		
Cor total	58.75	16			

α *Degree of freedom*.

* *Significant at 0.05 level*.

** *Significant at 0.01 level*.

**Figure 2:**
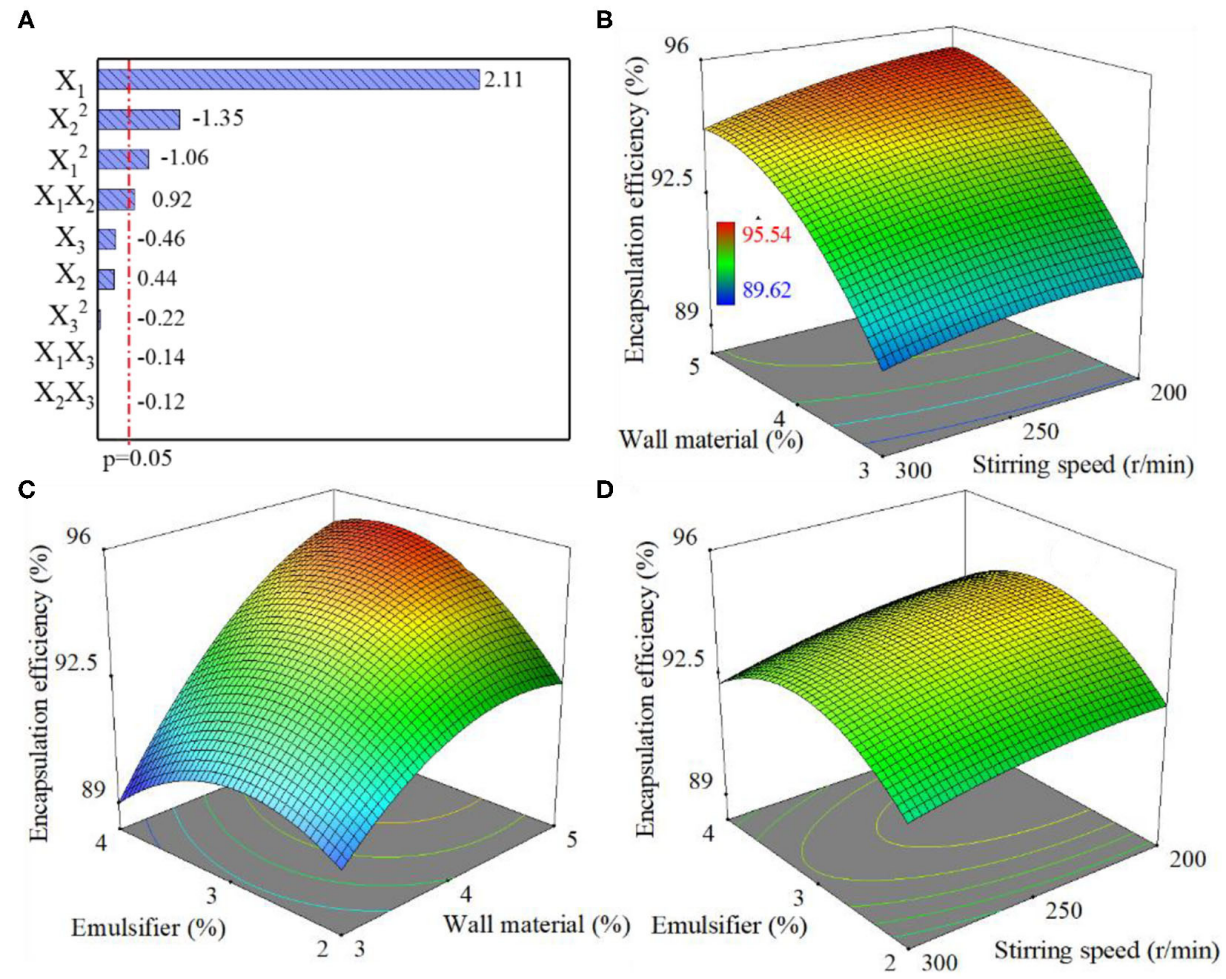
Pareto chart on the standardized effects of independent variables and their interaction on the encapsulation efficiency **(A)**. Response surface graphs of encapsulation efficiency when the dosage of emulsifier was 3% **(B)**, stirring speed was 250 r/min **(C)**, and the dosage of wall material was 4% **(D)**.

A pareto chart was plotted to illustrate the standardized effects of the independent variables and their interactions on encapsulation efficiency, where the length of each bar represents the standardized effect of each factor on the response (Solanki et al., 2007). The coefficients of X_1_, X_2_, and X_3_ denoted as the main effects on the EE and represent the average result of changing 1 variable at a time from its low level to its high level. The interaction terms (X_1_X_2_, X_1_X_3_, X_2_X_3_, ${\text{X}}_{1}^{2}$, ${\text{X}}_{2}^{2}$, and ${\text{X}}_{3}^{2}$) display how the EE changes when 2 variables are simultaneously changed. The positive coefficients for independent variables (X_1_ and X_2_) indicate the increase in wall-material and emulsifier dose cause a favorable effect on the EE, while the negative coefficients for the interactions between 2 variables (X_1_X_3_, X_2_X_3_, ${\text{X}}_{1}^{2}$, ${\text{X}}_{2}^{2}$, and ${\text{X}}_{3}^{2}$) indicate an unfavorable effect on the EE. Among the 3 independent variables the largest coefficient value is for X_1_ (b_1_ = 2.11 and *P* < 0.05), and the interaction terms X_1_X_2_, ${\text{X}}_{1}^{2}$, and ${\text{X}}_{2}^{2}$ exhibit coefficient value with *P* < 0.05 indicating that the variable is significant in the prediction of the EE. As depicted in Figure 2A, the bar for X_2_, X_3_, X_1_X_3_, X_2_X_3_, and ${\text{X}}_{3}^{2}$ remains inside the reference line, indicating these terms contribute little in the prediction of EE. Hence, these terms are omitted from the complete model to obtain a reduced second-order polynomial equation, and the resulting model is shown in Equation 6:

(6) $$\begin{array}{l} \text{EE}\left(\%\right)\;=\;94.01+2.22{\text{X}}_{1}+0.92{\text{X}}_{1}{\text{X}}_{2}-1.06{\text{X}}_{1}^{2}-1.35{\text{X}}_{2}^{2} \end{array}$$

### Response Surface Analysis of the Impact of Process Variables on EE

Response surface analysis was conducted to investigate the effects of process variables on EE. According to Equation 6, high wall material dosage indicates a positive linear effect (X_1_) and negative quadratic influence (${\text{X}}_{1}^{2}$), and also displays positive interactions with an emulsifier (X_1_X_2_) on the response. Namely, high wall material dosage can be beneficial to achieving higher EE, but due to the negative quadratic term, this effect can be lowered. As shown in Figure 2B, in all range of stirring speed, the larger the wall material dose, the higher the EE. As shown in Figure 2C, at a low emulsifier dosage, the response EE showed a tendency of first increasing and then decreasing with the growth of wall material dosage (Bouriche et al., 2019). This is mainly due to the lack of emulsifiers, which led to the failure of stabilizing the O/W emulsion, in turn, reducing the polymerization of isocyanate at the interface and resulting in inhibition of microcapsule formation. Considering positive interactions with an emulsifier, the negative effect did not appear at high emulsifier dosage, which is probably because the amount of emulsifier became less significant.

As shown in Equation 6, the emulsifier dosage had a relatively high negative quadratic influence (${\text{X}}_{2}^{2}$) and positive interactions with wall material dosage (X_1_X_2_) on the response EE. As shown in Figure 2B, under certain conditions, the higher the dosage of emulsifier in the organic phase, the lower the EE, which is due to the negative quadratic influence of emulsifier dosage. As shown in Figure 2C, an excessively high emulsifier dosage plus low wall material dosage was unfavorable to realizing high EE. This is mainly because insufficient wall material dosage resulted in fewer microcapsules to pack pretilachlor into PMS. When there was an excessively high emulsifier dosage in the organic phase, the EE monotonously increased with the increase of wall material dosage. This is probably due to abundant emulsifier that enhanced the stability between the organic and aqueous phase (Almeida et al., 2019; Bouriche et al., 2019). Furthermore, when the wall material dosage was high, the EE monotonously increased with the increased emulsifier dosage, which was most likely due to the increased stabilization effect of the emulsifier on the W/O phase, which is beneficial for the polymerization of isocyanate at the interface, facilitating microcapsule wall formation. As shown in Figures 2B,D, there was an insignificant change of EE in all range of stirring speed, which is consistent with other results that indicate that the stirring speed contributes little in the prediction of the EE.

### Model Adequacy

**Figure 3:**
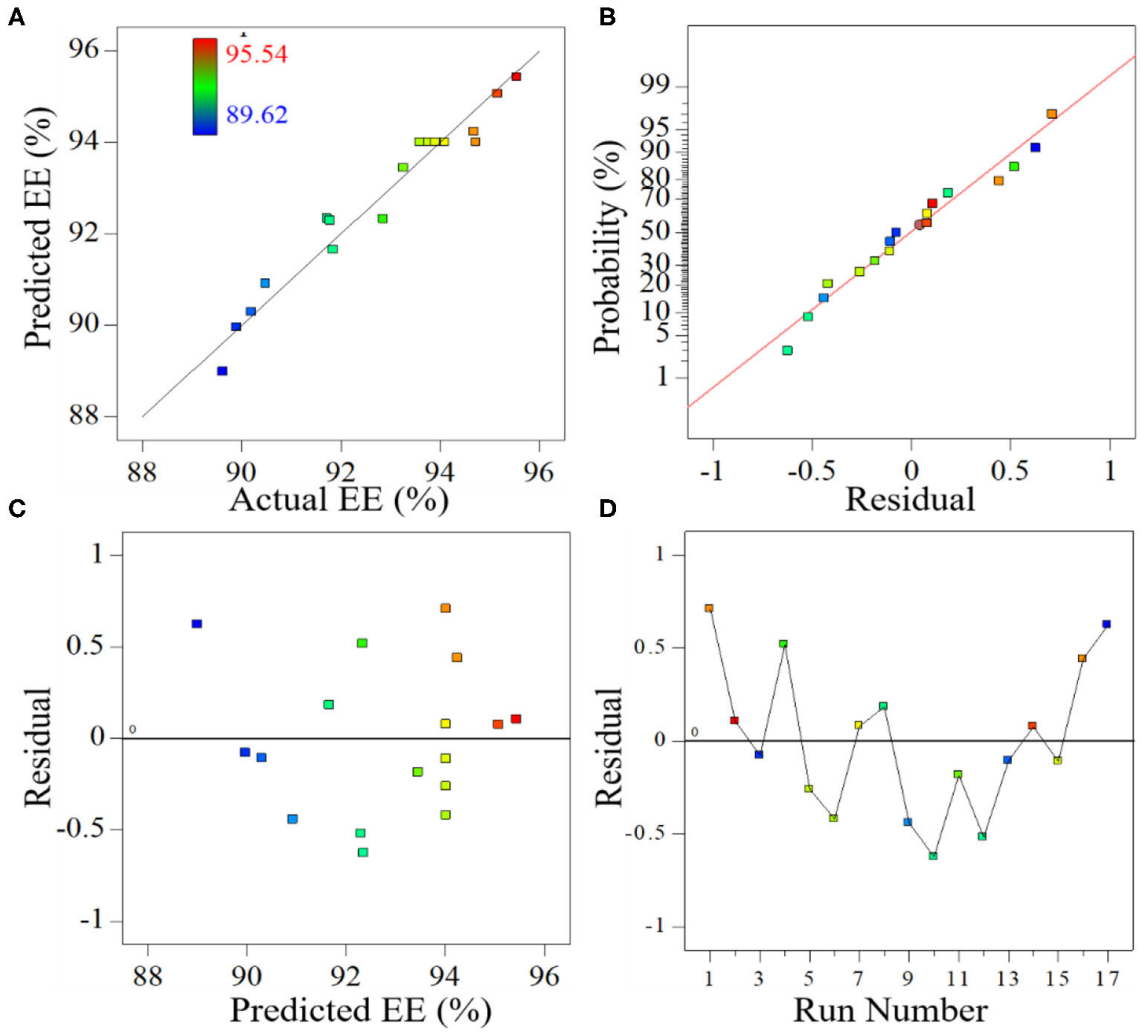
Relation between predicted value and observed values of encapsulation efficiency **(A)**, normal (percent) probability of residuals for encapsulation efficiency **(B)**, a residual plot of the predicted encapsulation efficiency **(C)**, and a residual plot of the run number **(D)**.

The relationship between the predicted values and the experimental values of EE were investigated to check the adequacy of the model. As shown in Figure 3A, the predicted values are quite close to the experimental values, and all the predicted points have a linear relation to experimental response values, indicating that the model developed in this study successfully captured the correlation between the variables and the response. Because the residuals represent the difference between the experimental value and the predicted value in the regression analysis, the normality of the data was checked based on a normal probability plot (NPP) of the residuals (Takahashi et al., 2008; Christian and Wagh, 2018). As shown in Figure 3B, the experimental points are reasonably aligned and fall near a straight line, suggesting a normal distribution. This description of the relationship between the residual and the predicted EE response indicates that the residuals are scattered randomly around zero (Figure 3C), meaning that the random errors follow a normal distribution. Moreover, as shown in the plot of residuals vs. experimental runs (Figure 3D), all the data points lie within the limits and are randomly scattered around zero, revealing that the regression terms correlate little with each other (Prakash Maran et al., 2013). Overall, it can be concluded that the proposed model is predicts the EE of PMS under given variables.

### Validation and Characterization of PMS

According to the optimal formula collected in this study, the highest standard of EE was set by the Design-Expert software to obtain optimal preparation parameters. It predicted preparation of PMS with wall material dosage of 5% and emulsifier dosage of 3.5% at polymerization stirring speed of 200 r/min, and an EE of 95.84%. Subsequently, three batches of PMS were prepared according to these desirable synthetic factors. The average EE value of the synthesized PMS was 95.68%, which reflects the predicted value (95.84%), proving that the regression equation model obtained by response surface design, has an accurate predictive ability.

**Figure 4:**
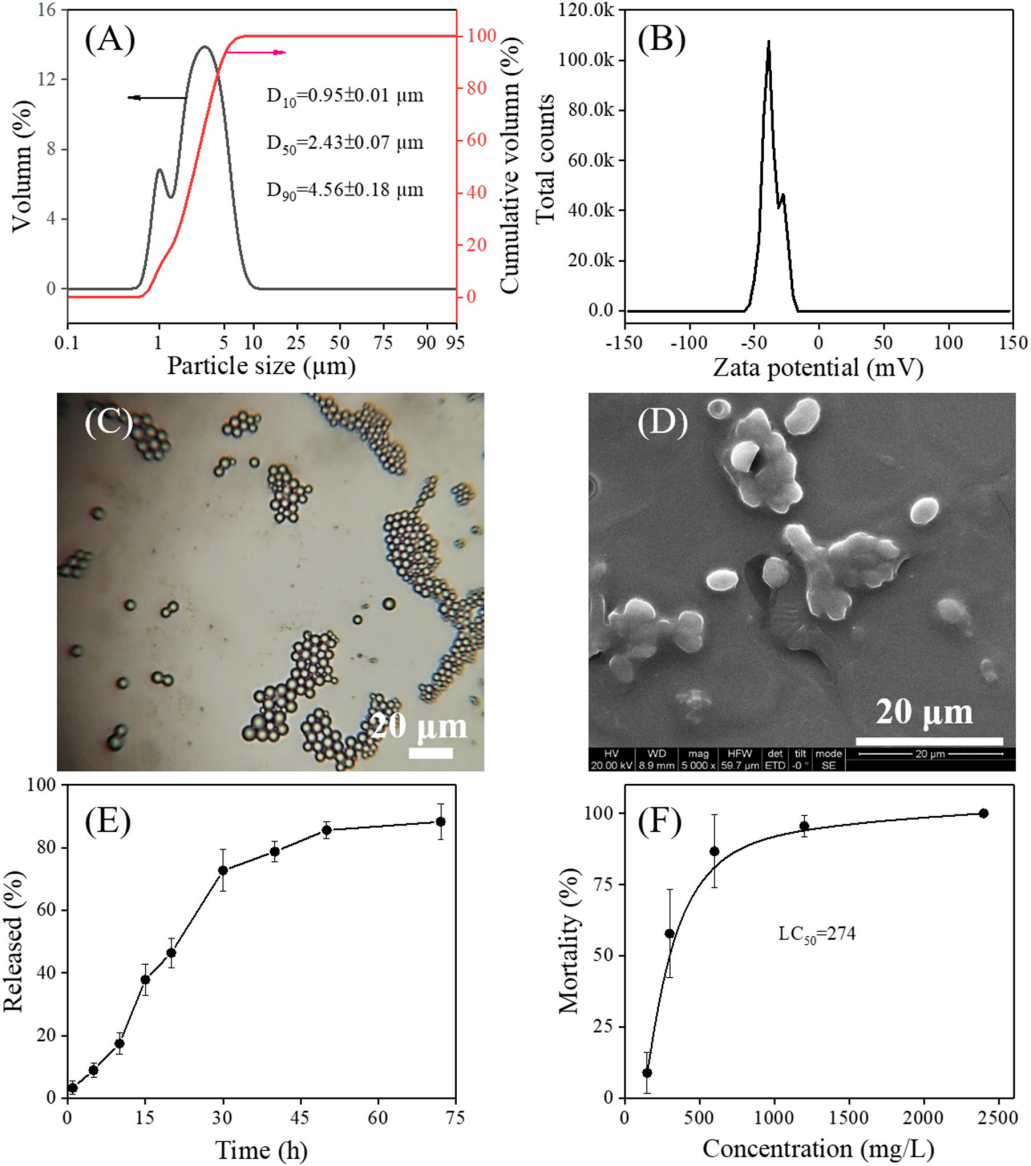
Particle size distribution **(A)**, Zeta potential **(B)**, an optical micrographic image at 10 × 40 magnification **(C)**, and scanning electron micrographs **(D)** of the as-prepared PMS. *In vitro* pretilachlor release curves of the prepared PMS incubated at acidic (pH = 4.0) conditions **(E)**. Barnyard grass mortalities after treated with different concentrations of PMS for 14 days **(F)**.

It is believed that the small size of microcapsules may allow for better transport of the herbicide through cell membranes, thereby resulting in enhanced efficacy (Hazra and Purkait, 2019). Considering the fact that the size of the microcapsule plays a critical role in governing the herbicide release rate (Mohanraj and Chen, 2006), the particle size and size distribution of PMS were measured by a laser particle size analyzer. As shown in Figure 4A, the narrow size distribution of PMS was found, covering approximately 0.7–10 μm, and the D_10_, D_50_, and D_90_ are measured to be 0.95 ± 0.01 μm, 2.43 ± 0.07 μm, and 4.56 ± 0.18 μm, respectively. The Zeta potential of PMS represents a negatively charged surface with a total value −36.8 ± 6.9 mV (Figure 4B), mainly due to the absorption of strong negatively charged adjuvant alkyl naphthalene sulfonate formaldehyde polymer onto the polyurea wall. It is worth noting that a particle charged with a potential of (±) 30 mV has proved to be stable in the suspension, owing to its repulsion capacity to prevent particle aggregation (Schaffazick et al., 2003; Mohanraj and Chen, 2006; Diyanat et al., 2019). In addition, physicochemical stability was investigated by measuring the pretilachlor contents and encapsulation efficiency of the PMS over a period of 14 days. As shown in Supplementary Table 1, the pretilachlor contents and encapsulation efficiency of the PMS differ insignificantly after being stored at low (0^o^C), normal (25^o^C), and high (55^o^C) temperatures, demonstrating the excellent storage stability of PMS. Afterward, as shown in Figure 4C, the naturally dried PMS exhibited a spherical shape with an average particle size of ~2.92 μm, which is close to the D_50_ particle size collected in the solution.

The surface morphology of the synthesized PMS was characterized using SEM microscopy. As shown in Figure 4D, single PMS particles have an ellipsoid shape and particle size range within 3–5 μm, which is similar to that collected from optical microscopy, indicating that the polyurea wall has sufficient strength to retain its shape. The change in shape from spherical to ellipsoid might contribute to the shell-determined elastic forces (Takahashi et al., 2008; Tang et al., 2012). Although parts of PMS particles overlap with each other, this could be due to the deposition of adjuvants such as xanthan gum and Tween-60, as no specific purification was performed during the preparation of the samples for SEM scanning.

### *In vitro* Pretilachlor Release Behavior and Herbicidal Activity of PMS

The drug release capacity of microcapsules is essential to giving full play to its function. By collecting the cumulative release of the pretilachlor as a function of time, the *in-vitro* pretilachlor release behavior of PMS was investigated in an acidic medium. As depicted in Figure 4E, PMS exhibited delayed release performance, about 85% of the drug was quickly released within 50 h, and then released slowly within 72 h. As illustrated in Scheme 1, the delayed release performance of PMS is mainly due to the degradation of the polyurea shell wall, because the reactive free primary amine groups of the polyurea shell wall generated soluble salt under acidic conditions, resulting in a high degree of pretilachlor release in the core (Hedaoo et al., 2013). The release kinetics were also elaborated using the Korsmeyer–Peppas mathematical model (Equation 3). The *n* value obtained for the release exponent was 0.7964 in the range of 0.45 < *n* < 0.85, indicating a combination of the coexistence of diffusion-controlled pretilachlor release and swelling-controlled pretilachlor release in the experimental medium (Ritger and Peppas, 1987a; Lee et al., 2013). Overall, the above results demonstrate that the PMS has delayed release performance, which can be used to minimize the adverse effects and environmental toxicity of pretilachlor in agricultural weeding.

The herbicidal activity of the PMS against barnyard grass was investigated. Barnyard grass was treated with different dosages of PMS. As shown in Figure 4F, the barnyard grass mortalities increased quickly at first and reached 100% as PMS dosage increased. The LC_50_ value was calculated to be 274 mg/L. The barnyard grass presented leaves toxicosis or leaves death as the dosage of PMS treatment increased, revealing the effective herbicidal capability of the as-synthesized PMS. In light of this practical application, it is necessary to provide a reference dosage of PMS to guarantee effectiveness while avoiding overuse. When treated with 150 mg/L of PMS, the barnyard grass exhibited average mortality of <10%, suggesting that this dose (150 mg/L of PMS) is insufficient for controlling weeds. However, once PMS dosage exceeded 1,200 mg/L, no barnyard grass survived, leading to nearly 100% mortality, indicating an overuse of PMS. Considering that the excessive use of herbicides would lead to mortality, the recommended guidance dose of PMS should be within 300–600 mg/L [or 270–540 g (a.i.)/ha], under which the average mortality ranged from 57.78 to 86.67%.

### Evaluation of PMS Practical Application

**Figure 5:**
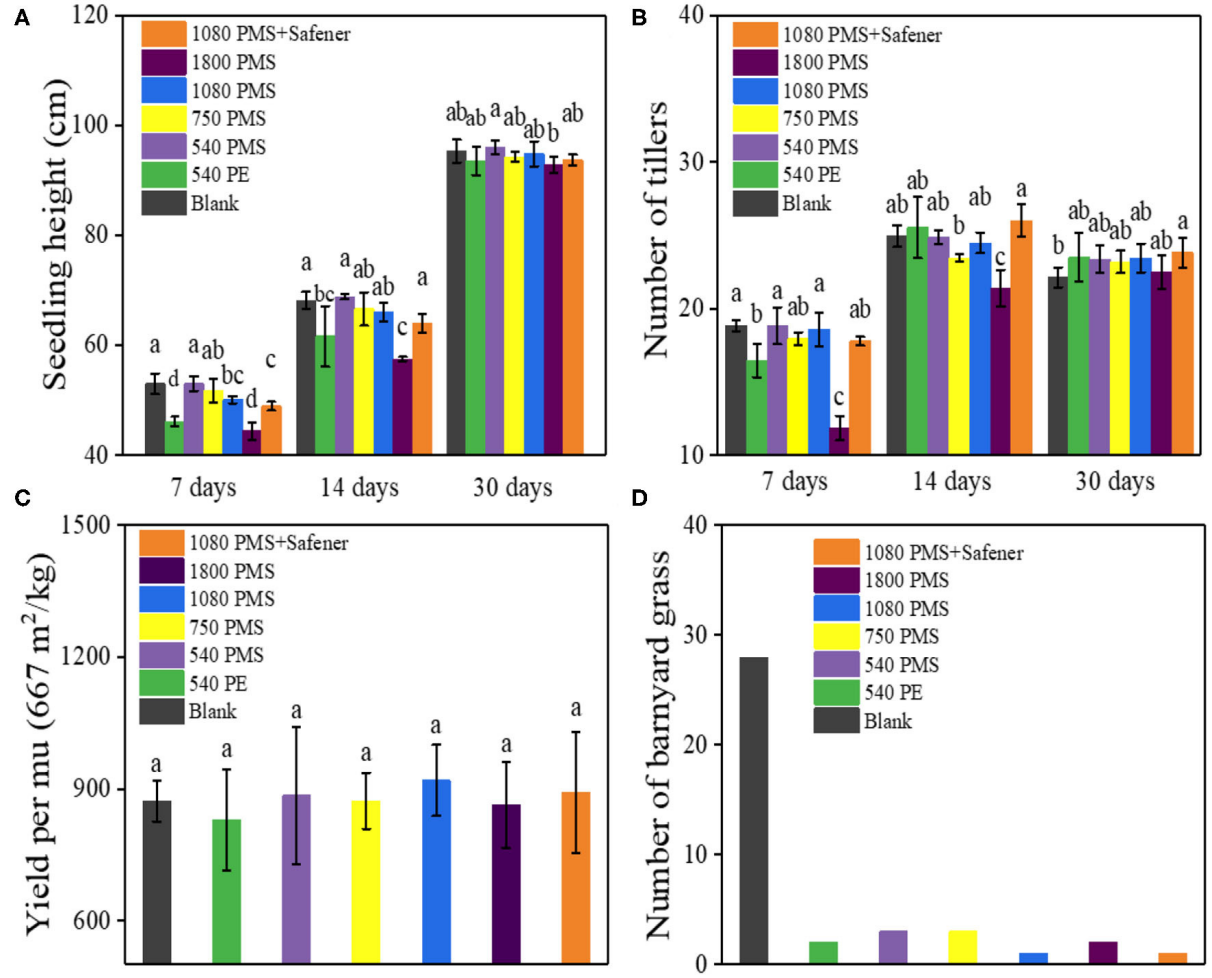
The results of rice growth in the paddy field after it was treated with different concentrations of PMS, PE, and PMS with safeners. The relative seedling height **(A)** and number of tillers **(B)** after treatment for 7, 14, and 30 days, respectively, and the corresponding harvest yield **(C)**. The number of barnyard grasses in the experimental paddy field **(D)** (Note: Data marked with different lowercase letters are significantly different at the *p* < 0.05 level by Duncan's multiple-range test).

The practical application and safety of PMS in weeding paddy fields were evaluated. We considered several morphophysiological factors such as the plant height, tiller number, rice yield, and measured a number of barnyard grasses after PMS treatment at different times. After 7 days of the low dose [540 and 750 g (ai)/ha] PMS treatment, rice plant height (purple and yellow column in Figure 5A) and tiller number (purple and yellow column in Figure 5B) did not change significantly, but high dose [1,080 and 1,800 g (ai)/ha] PMS treatment resulted in significant inhibition of such two indexes. Fortunately, the inhibition effect was relieved by adding safeners (orange column in Figures 5A,B). The inhibition rate of 1,080 g (ai)/ha PMS treatment on rice plant height (5.45%, blue column in Figure 5A) and tillers number (1.50%, blue column in Figure 5B) was lower than that of commercial available PE treatment on rice plant height (12.89%, green column in Figure 5A), and tillers number (12.66%, green column in Figure 5B). After treated for 14 days, except for high PMS dosage [1,800 g (ai)/ha] treatment, which generated a significant inhibition effect on rice plant height and tillers number. Other treatments exerted insignificant inhibition effects on rice plant height and tiller number. In comparison, the inhibition rate of 1,080 g (ai)/ha PMS treatment on rice plant height decreased from 16.69 to 16.00%, while the inhibition rate of tillers number was decreased from 39.02 to 15.19%. After 30 days of treatment, the rice growth (including the plant height and tillers number) restored under all PMS treatments, indicating the inhibitory effect on rice growth induced by PMS can be repaired in practical weeding. The recovery of rice growth was mainly due to the widely reported self-repairing effect of rice (Liu et al., 2018; Sun et al., 2019). As shown in Figure 5C, the difference in the yield of all PMS-treated rice was not obvious. In particular, the yield of PMS-treated rice was equal to the yield of 30% PE-treated rice, suggesting that the PMS has promising potential applications in the weed management of transplanted rice. The number of barnyard grasses also significantly decreased after PMS treatment (Figure 5D). The as-synthesized PMS, when used for barnyard grass removal in rice fields, has little effect on the yield of rice, meaning it has excellent prospects and potential applications.

### Pretilachlor Formulation Comparison

Through the interfacial polymerization reaction, preparachlor microcapsules were prepared with different types of polymers such as polyurea, polyethylene glycol (PEG), and polycaprolacton (Kumar et al., 2016; Christian and Wagh, 2018; Diyanat et al., 2019). Therefore, the synthesized PMS was compared with other reported pretilachlor microcapsule formulations, with some parameters listed in Supplementary Table 2. As illustrated in Supplementary Table 2, the organic solvents were used in all types of formulation methods, but the series of solvents differ greatly. It is worth noting that a higher boiling and flash point is beneficial to reduce the risk of solvent evaporation and explosion (Sun et al., 2019). In this regard, the synthetic procedure for PMS is more conducive to the practical production of herbicide formulation as a high boiling point (bf) and flash point (fp) solvent was used. The pretilachlor encapsulation efficiency of PMS was also higher than that of polyurea microcapsule synthesized with HMDI and HMDA, which is beneficial in declining the total amount of organic adjuvants. Moreover, PMS prepared by isocyanate polymerization does not need extra organic chain extenders, which is conducive to more economical synthesis and able to reduce organic pollution to the environment.

## Conclusions

In conclusion, this study prepared a pretilachlor microcapsule suspension formulation with high EE using the interfacial polymerization technique. A Box–Behnken response surface design was successfully used to investigate the individual and joint effect of process variables (such as wall material dosage, emulsifier dosage, and the polymerization stirring speed) on the EE of PMS. The developed regression equation model predicted the EE of PMS. Results showed that the dosage of wall materials and emulsifiers could significantly affect the maximum EE of PMS. The optimum conditions for PMS preparation included a 5% wall material dose, 3.5% emulsifier dose at a polymerization stirring speed of 200 r/min, under which the maximum EE was expected to be 95.84%. Specifically, the practical EE values of PMS obtained under optimal experimental conditions agreed well with the predicted values. In addition, PMS has proven to delay release capability and *in vivo* herbicidal activity against barnyard grass, with an LC_50_ value of 274 mg/L. Furthermore, PMS was proved to have a higher potential in practical weed management compared to commercially available PE and could be applied to weeding paddy fields.

## Data Availability Statement

The raw data supporting the conclusions of this article will be made available by the authors, without undue reservation.

## Author Contributions

HC and XL conceived the study. HW, SD, LH, and HC performed all the experiments. HC wrote and critically edited the manuscript. JL, CJ, and XO revised the manuscript. All authors contributed to the article and approved the submitted version.

## Conflict of Interest

The authors declare that the research was conducted in the absence of any commercial or financial relationships that could be construed as a potential conflict of interest.
