# Supplementary material for: Pretilachlor Releasable Polyurea Microcapsules Suspension Optimization and Its Paddy Field Weeding Investigation
Source: Front Chem. 2020 Oct 22;8:826. doi: 10.3389/fchem.2020.00826 (PMC7642302; doi:10.3389/fchem.2020.00826)
Supplement: Supplementary file 1 [file Data_Sheet_1.PDF]

Supporting information

Pretilachlor releasable polyurea microcapsules suspension optimization and its paddy field weeding investigation

Hongjun Chen[1], Xiu Liu[2]*, Siyu Wu[2], Hongkun Wang[2, 3], Xiaoming Ou[4], Linya Hang, Jingbo Li, Chenzhong Jin[2]*

[1] Hunan Provincial Key Laboratory of Fine Ceramics and Powder Materials, School of Materials and Environmental Engineering, Hunan University of Humanities, Science and Technology, Loudi, 417000, P. R. China

[2] Key Laboratory of Pesticide Harmless Application, Collaborative Innovation Center for Field Weeds Control (CICFWC) of Hunan Province, Hunan University of Humanities, Science and Technology, Loudi, 417000, P. R. China

[3] Forestry Bureau of Lanshan County, Lanshan, 425800, P. R. China

[4] National Engineering Research Center for Agrochemicals, Hunan Research Institute of Chemical Industry, Changsha 410007, P. R. China

*E-mail: liuxiu841027@163.com (X. Liu); hnldjcz@sina.com (C.Z. Jin)

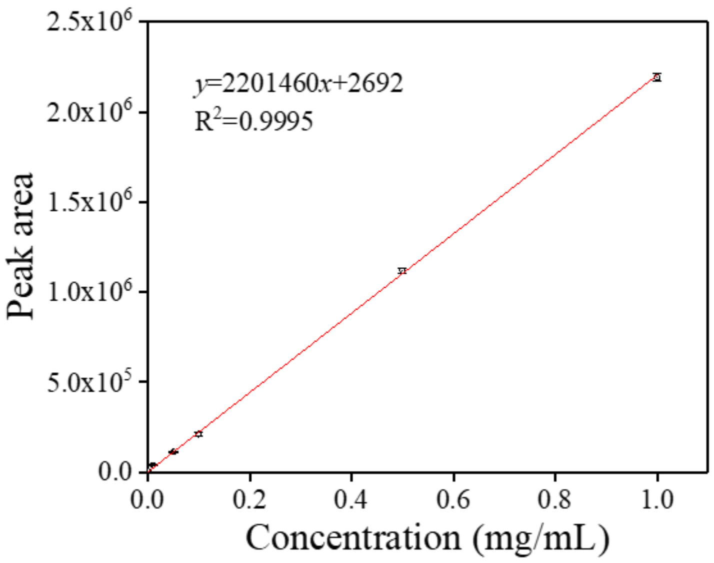

Figure S1. Calibration curves plotted by spiked pretilachlor samples.

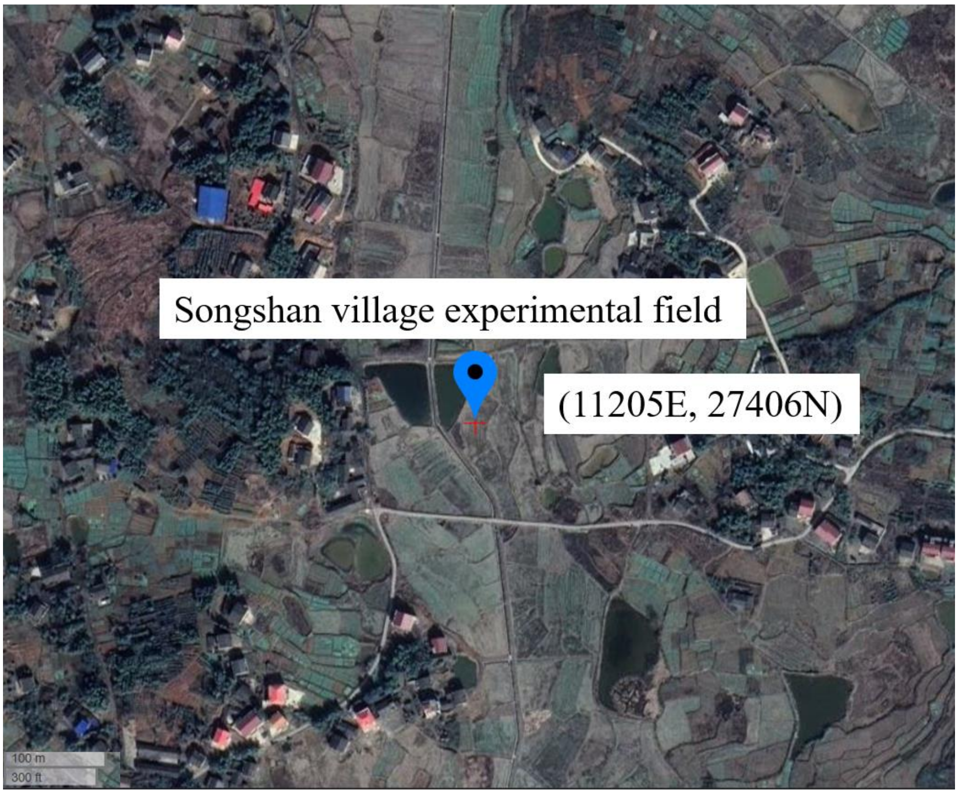

Figure S2. Satellite location of the experimental field trials

Table S1. Results of pretilachlor content and encapsulation efficiency of PMS packed in glass tubes at 25and stored at 0 ºC, 25 ºC and 55 ºC, respectively. (Note: Data marked with different lowercase letters are significantly different at the $p < 0.05$ level by Duncan's multiple-range test.)

Storage temperature	Pretilachlor content (%)		EE (%)	
	Original	After 14 day	Original	After 14 day
25±1 ºC	24.58±0.36	24.32±0.36a	95.58±0.29	95.33±0.58a
0±3 ºC	24.58±0.36	24.02±0.41a	95.58±0.29	95.12±0.69a
55±1 ºC	24.58±0.36	23.85±0.23a	95.58±0.29	94.92±0.84a

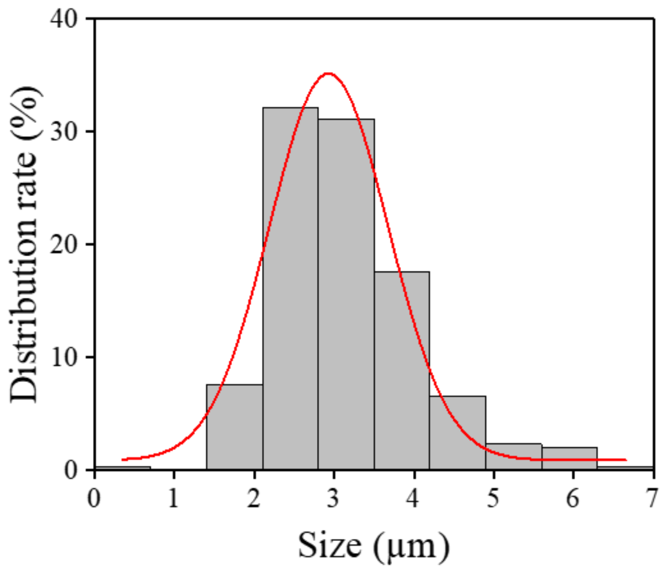

Figure S3. The size distribution of PMS derived from the optical micrographic image of Figure 4C.

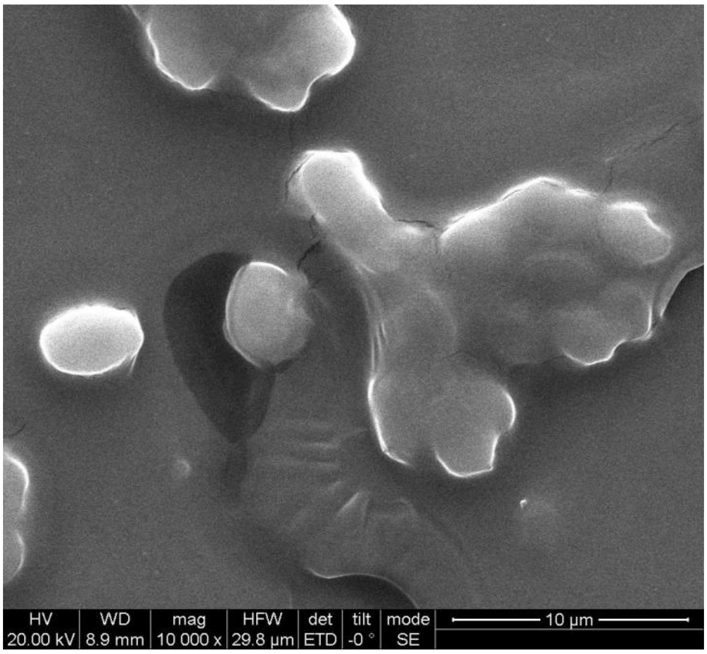

Figure S4. Amplified scanning electron micrograph of the as-prepared PMS according to Figure 4D.

Table S2. Comparison of the polyurea pretilachlor PMS with other previously-reported pretilachlor formulation synthesized by interfacial polymerization method.

Formulation	Polymerization contents	Adjuvant	Solvent	Particle size	Pretilachlor EE (%)	Dosage (g/ha)	Ref.
Polycaprolactone nanocapsules suspension	poly ε-caprolactone	oil, Span 60, Tween 80,	Acetone bp: 56.12 ºC fp: -17.8 ºC	199 ± 9 nm	99.5 ± 1.3	1500	[3]
Polyethylene glycol (PEG) encapsulated nanospheres	PEG-1500 +adipic acid	no	Dichloromethane bp: 39.8 ºC fp: 34 ºC	1-100 nm	Not mentioned	300-600	[19]
Polyurea microcapsule	HMDI+HMDA	Tween-85	n-Octane bp: 125.6 ºC fp: 13 ºC	Average 3.2 µm	90.78	-	[23]
Polyurea microcapsule suspension	PM-200	MF, Tween 60, xanthan gum	S-200 bp: 227-287 ºC fp: 106 ºC	D_{90} 4.56±0.18 µm	95.58	540-1080	This paper

bp: represents the boiling point;
fp: represents the flash point
